# Collecting behavioural data across countries during pandemics: Development of the COVID-19 Risk Assessment Tool

**DOI:** 10.3758/s13428-025-02743-x

**Published:** 2025-07-14

**Authors:** Gjalt-Jorn Peters, Dominika Kwasnicka, Gill A. ten Hoor, Rik Crutzen, Tugce Varol, Lisa Marie Warner, Mahdi Algargoosh, Eskinder Eshetu Ali, Mudassir Anwar, Sali Rahadi Asih, Zuhal Feryal Baltas, Emma Berry, Kebede Beyene, Katarzyna Anna Campbell, Bruno Moreira Carneiro, Laura Castillo-Eito, Amy Hai Yan Chan, Samuel Suk-Hung Chan, Sabrina Cipolletta, Ann DeSmet, Triana Kesuma Dewi, Alexandra Lelia Dima, Jorge Encantado, Tracy Epton, João Figueiredo, Gustavo DalCin Fracaroli, Aurelie Gauchet, Gebremedhin Beedemariam Gebretekle, Pierre Gérain, Cristina Albuquerque Godinho, Lisa Graham-Wisener, James A. Green, Jenny M. Groarke, Thomas Gültzow, Elif Basak Guven, Roel C. J. Hermans, Sander Hermsen, Jennifer Inauen, Angelos P. Kassianos, Tatiana Valerievna Kazantseva, Els Keyaerts, Laura Maria König, Daniela Lange, Emelien Lauwerier, Yongchan Lie, Andrian Liem, Aleksandra Luszczynska, Marta M. Marques, Hannah Catherine Moore, Chris Noone, Johanna Nurmi, Ratri Nurwanti, Elif Suna Ozbay, Iga Palacz-Poborczyk, Rebecca Anne Pedruzzi, Louise Poppe, Lucy Mabel Porter, Daniel Powell, Bruna Salati Nan Rinaldi, Alexis Ruffault, Carsten Schmitz, Urte Scholz, Ana-Maria Schweitzer, Yasemin Selekoğlu Ok, Medha Shree, Carolina C. Silva, Yasinta Astin Sokang, Albert W. Tam, Mei Yee Tang, Silvia Caterina Maria Tomaino, Samantha Barbara van Beurden, Stefan Verweij, Stan Vluggen, Rochelle E. Watkins, Szilvia Zörgő, Sylvia Roozen

**Affiliations:** 1 Open University of the Netherlands, Heerlen, Netherlands; 2 University of Melbourne, Parkville, Australia; 3 Maastricht University, Maastricht, The Netherlands; 4 MSB Medical School Berlin, Berlin, Germany; 5 The University of Auckland, Auckland, New Zealand; 6 King Faisal Specialist Hospital and Research Centre, Riyadh, Saudi Arabia; 7 Addis Ababa University, Addis Ababa, Ethiopia; 8 University of Otago, Dunedin, New Zealand; 9 Universitas Indonesia, Depok, Indonesia; 10 Cerrehpaşa University Faculty of Medicine, İstanbul, Turkey; 11 Queen’s University Belfast, Belfast, UK; 12 University of Nottingham, Nottingham, UK; 13 Universidade Federal de Rondonópolis, Rondonópolis, Brazil; 14 University of Gloucestershire, Cheltenham, UK; 15 University of Padua, Padova, Italy; 16 Université libre de Bruxelles, Bruxelles, Belgium; 17 University of Antwerp, Antwerp, Belgium; 18 Airlangga University, Surabaya, Indonesia; 19 Avedis Donabedian Research Institute (FAD), Barcelona, Spain; 20 Universitat Autònoma de Barcelona (UAB), Barcelona, Spain; 21 Avaluació de tecnologies sanitàries en atenció primària i salut mental (PRISMA), Institut de Recerca Sant Joan de Déu (IRSJD), Esplugues de Llobregat, Spain; 22 Consortium “Centro de Investigación Biomédica en Red” Epidemiology and Public Health (CIBERESP), Madrid, Spain; 23 Universidade de Lisboa, Lisboa, Portugal; 24 University of Manchester, Manchester, UK; 25 Instituto Superior Técnico, Lisbon, Portugal; 26 SAST - Segurança e Saúde do Trabalho, São Bernardo do Campo, Brazil; 27 University Savoie Mont Blanc, Chambéry, France; 28 University of Toronto, Toronto, Canada; 29 Universidade Católica Portuguesa, Lisboa, Portugal; 30 University of Limerick, Limerick, Ireland; 31 Istanbul Technical University, İstanbul, Turkey; 32 LeefstijlLab Arnhem, Arnhem, Netherlands; 33 Radboud University Medical Centre, Nijmegen, Netherlands; 34 University of Bern, Bern, Switzerland; 35 Cyprus University of Technology, Limassol, Cyprus; 36 University College London, London, UK; 37 Saint-Petersburg State University, St Petersburg, Russian Federation; 38 UZ Leuven, Leuven, Belgium; 39 University of Vienna, Vienna, Austria; 40 Universitas Sebelas Maret, Surakarta City, Indonesia; 41 SWPS University, Warszawa, Poland; 42 Trinity College Dublin, Dublin 2, Ireland; 43 National School of Public Health, Comprehensive Health Research Centre, NOVA University of Lisbon, Lisbon, Portugal; 44 The Kids Research Institute Australia, Perth, Australia; 45 University of Galway, Galway, Ireland; 46 University of Helsinki, Helsinki, Finland; 47 University of Cambridge, Cambridge, UK; 48 Universitas Brawijaya, Malang, Indonesia; 49 Bilkent University, Ankara, Turkey; 50 The Kids Research Institute, University of Western Australia, Perth, Australia; 51 Ghent University, Ghent, Belgium; 52 University of Exeter, Exeter, UK; 53 University of Aberdeen, Aberdeen, UK; 54 Holy House of Mercy of Piracicaba, Piracicaba, Brazil; 55 French Institute of Sport (INSEP), Paris, France; 56 LimeSurvey GmbH, Hamburg, Germany; 57 University of Zurich, Zürich, Switzerland; 58 Fundatia Baylor Marea Neagra, Constanța, Romania; 59 Dokuz Eylül University, İzmir, Turkey; 60 Viztar International, Navi Mumbai, India; 61 Krida Wacana Christian University, West Jakarta, Indonesia; 62 Fastgen Corporation, San Mateo, USA; 63 University of Leeds, Leeds, UK; 64 University of Groningen, Groningen, Netherlands; 65 Zuyd University of Applied Sciences, Heerlen, Netherlands; 66 The University of Western Australia, Perth, Australia; 67 Université Grenoble Alpes, Grenoble, France

**Keywords:** Health behaviour change, Impact, Risk, People, Strategies, Systems, COVID-19

## Abstract

Tools that can be used to collect behavioural data during pandemics are needed to inform policy and practice. The objective of this project was to develop the *Your COVID-19 Risk* tool in response to the global spread of COVID-19, aiming to promote health behaviour change. We developed an online resource based on key behavioural evidence-based risk factors related to contracting and spreading COVID-19. This tool allows for assessing risk and provides instant support to protect individuals from infection. The Risk Estimation Questions assessed users’ location, age, gender, work environment, day-to-day behaviours currently performed, and conditions under which these behaviours would change. Users were also asked to estimate how often they keep their distance from others in public and regularly wash their hands, and the procedures they follow to do so. A multidisciplinary research team of more than 150 international experts developed the tool. Over 60,000 users in more than 150 countries have assessed their risk and provided data. The majority of respondents reported that they almost always keep their distance from others in public places, and most participants reported washing their hands after touching public or shared surfaces or when entering buildings. The tool, data, and results were openly shared to support government and health agencies developing behaviour change interventions. This tool creates a blueprint for similar digital infrastructure that can be replicated and used in future pandemics.

**Supplementary Information:**

The online version contains supplementary material available at 10.3758/s13428-025-02743-x.

## Introduction

The COVID-19 outbreak has substantially increased the burden on healthcare systems (Cong et al., 2024; Metersky et al., 2024) through increased primary care, outpatient, and emergency department presentations due to the virus itself and through indirect mechanisms such as increasing rates of poor mental health (Alimoradi et al., 2024; Orban et al., 2024). In the absence of widely available treatments and vaccines, which only became available in most countries in 2021, and are now well researched and widely available (Li et al., 2023), rapidly rising prevalence and mortality rates have forced most countries to introduce various preventive and protective measures, such as lockdowns, to slow the spread of the virus (Chiesa et al., 2021; Fuss et al., 2021; Oraby et al., 2021). Such measures have substantially disrupted social and economic systems around the world, globally impacting almost all countries and territories (Canwat, 2024; Delardas et al., 2022; Naseer et al., 2023).

Given the crucial role of human behaviour in the spread of the virus (Perofsky et al., 2024; West et al., 2020), most countries introduced preventive measures that require behaviour change, including social distancing, wearing masks, and increasing the frequency of hand washing (Ingram et al., 2021; Van Bavel et al., 2020). Globally, governments and health agencies have worked collaboratively with social scientists to influence human behaviour to slow down and to contain the virus spread (Bonell et al., 2020; Byrne-Davis et al., 2022; Van Bavel et al., 2020). The effectiveness of these behavioural policies has varied across countries and territories, with some achieving great success and others facing the challenges of new outbreaks (Bicchieri et al., 2021; Zhang, 2020).

Following the principles of Open Science (Kwasnicka et al., 2021), global cooperation, planning, and effective governance (Beyene et al., 2021), the scientists involved in this project joined forces to find solutions to prevent and contain the spread of SARS-CoV-2 (Sachs et al., 2020). The ‘Your COVID-19 Risk’ project commenced in March 2020, before SARS-CoV-2 vaccines were available, and at a time when the effectiveness of preventive measures was being debated. The project employed several technologies and methods to (1) facilitate the use of behavioural science solutions in a systematic manner, (2) develop and implement a tool that assesses behaviour-related COVID-19 risk, (3) provide instant risk estimates and suggestions for behaviour change, and (4) share the data with researchers, governments, and health agencies to inform future policy and practice during the pandemic. Responding to the COVID-19 pandemic, the aims of this project were as follows:

1. To develop and provide a widely available tool that allows people to assess and understand their COVID-19-related risk, both to themselves and to others
2. To then apply behaviour change theory to present people with a brief intervention including persuasive messages that help them reduce their risk
3. To explore tool users’ COVID-19-related behaviours and related determinants in order to enable improvements to the tool
4. To recommend avenues for interventions and policy to governments, policymakers, and prevention and health promotion organisations

## Methods

### Project overview

This project applied insights from behaviour change science to develop, implement, and disseminate an online tool to help contain the COVID-19 pandemic for communities worldwide. This tool allows the user to assess the ‘additional risk’ of contracting and spreading the virus that leads to COVID-19. *Additional risk* involves situations and behaviours over which the individual has some control—and can do something about. The tool does not account for risks related to underlying health conditions. The tool development followed a rigorous, systematic, and scalable approach. The tool was created in ten weeks, in a worldwide, largely decentralised collaboration of over 150 experts. We (i.e., the group of volunteer experts who developed this tool) used theory and already existing empirical evidence. Below we describe elements of the tool and how it was developed, including development of a risk model, based on risk estimate, a brief intervention—tailored messages aimed at reducing their risk, and a set of questions that allow us to improve the intervention.

### Design

The Your COVID-19 Risk tool consists of 10 questions informed by a rapid systematic assessment of the literature addressing the risk factors for contracting and spreading SARS-CoV-2, and two expert consultations. We leveraged acyclic behaviour change diagrams (ABCDs; Peters & Crutzen, 2021) to develop and make transparent a behaviour change intervention that tool users received based on their answers to the questions. ABCDs are diagrams that illustrate the logic model, i.e., ‘theory of change’, underlying the intervention to change some aspect of perceptions and/or behaviours. Specifically, the ABCD shows the assumed causal and structural assumptions, demonstrating what is assumed to cause what (e.g., which elements of the intervention are assumed to influence which aspects of the target individual’s psyche) and what is assumed to consist of what (e.g., which determinants are assumed to contain which aspects of the target individual’s psyche). ABCDs are generated from a uniform, machine-readable format, including a description of behaviour change principles (BCPs), conditions for effectiveness, applications, determinants and sub-determinants, behaviours, and sub-behaviours (for more details and an example, see Metz et al., 2022).

Behavioural determinants (specifically, psychological constructs defined in the reasoned action approach, RAA; Fishbein & Ajzen, 2010) were specified using Decentralized Construct Taxonomies (Peters & Crutzen, 2024) to facilitate standardisation of construct definitions and corresponding measurement instruments across countries and contexts. Questions measuring these determinants were integrated into the tool, and the results of these questions were analysed using Confidence Interval-Based Estimation of Relevance (CIBER) plots to identify the most important determinants (Crutzen et al., 2017; Peters & Crutzen, 2018).

### The Risk estimate

To provide information about COVID-19-related risk, we first required a risk model that would estimate the risk based on the unique situations of tool users. That risk model was established in two expert consultations. The main goal of the first consultation was to determine the most prominent risk behaviours and risk factors. In order to provide tool users with an engaging tool, and to keep the number of questions to a minimum, we aimed to identify key behavioural factors that are within the users’ control and can be addressed through behaviour change principles. In the second expert consultation, we then determined the degree to which each risk behaviour and risk factor contributed to the overall risk estimate presented to tool users. The expert consultations and considerations underlying the ultimate risk estimate presentation are discussed in detail in the appendix ‘Your COVID-19 Risk: Technical background’ (Supplementary Material 1).

The risk estimate was determined to be due to three factors: ‘risk of getting the virus’ (i.e., through proximity or lack of social isolation), ‘risk from not removing the virus’ (i.e., through lack of handwashing), and other uncontrollable factors (e.g., demographics). A visual image combining these three factors was considered appropriate to communicate the risk level (e.g., an avatar of someone surrounded by virus) and a scale demonstrating the risk.

**Fig. 1:**
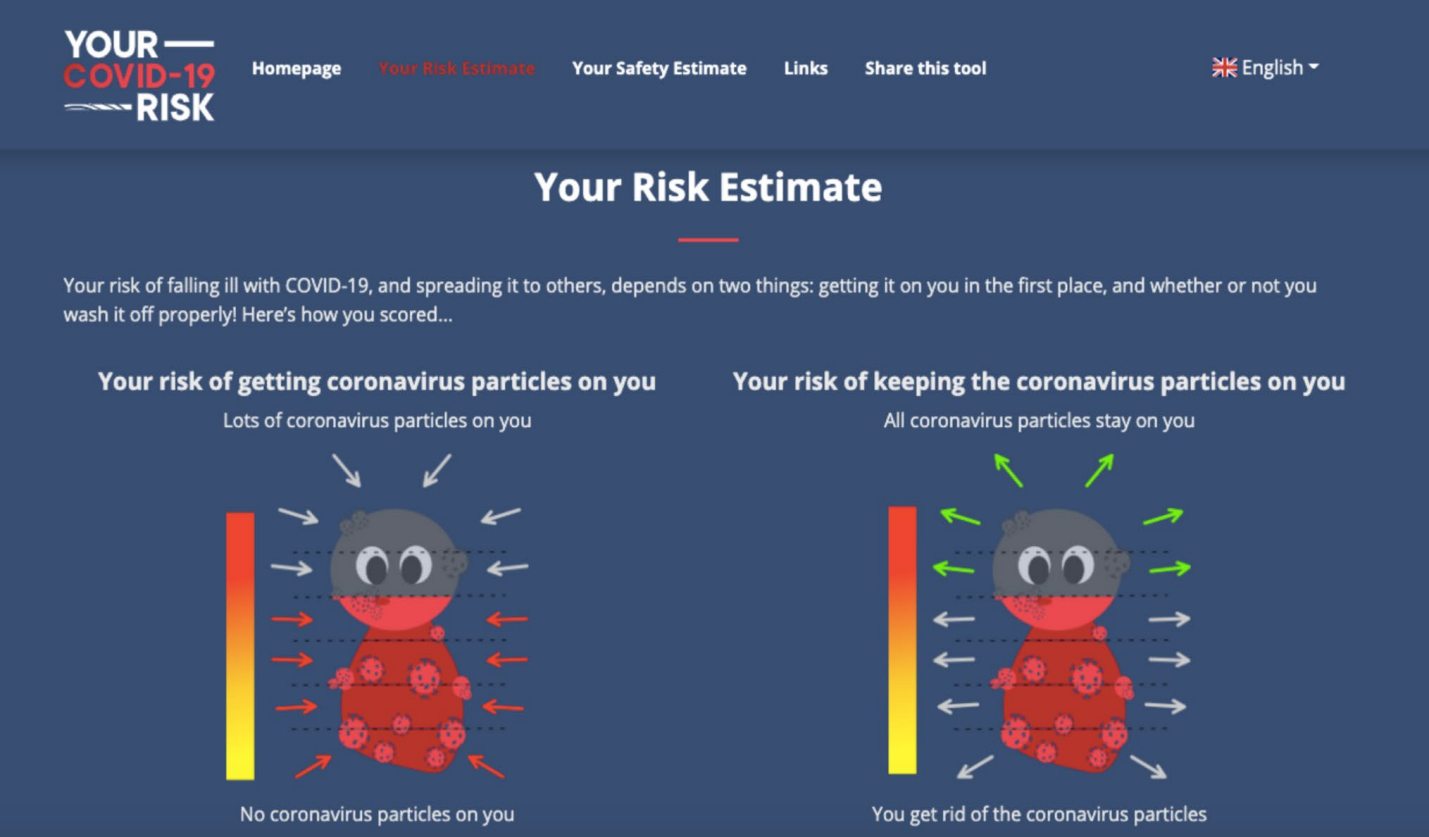
An example of the graphic representation of risk estimates

The design choices were discussed within the larger team of expert volunteers, and it was decided that one image for each type of risk would be presented, with a scale to indicate risk category (Fig. 1). The proposed materials were piloted with representatives of the general population to ensure that they were easy to understand and interpret. Subsequently, the materials were adjusted in line with the received feedback.

### The intervention

Along with the risk estimate, tool users were presented with a set of tailored messages aimed at reducing their risk. These messages addressed the three behaviours measured in the risk estimation questions: social isolation, distance keeping, and hand washing. As there were no determinant studies to guide the development of the tool, these messages were based on expert opinions about which determinants would be important for these three target behaviours. These determinants were then specified in an Acyclic Behaviour Change Diagram and matched to behaviour change methods appropriate for each determinant. This yielded a set of persuasive texts, a selection of which was based on participants’ reported behaviour and risk status. Details about this process, the underlying reasoning, and the acyclic behaviour change diagrams produced can be found in the corresponding Open Science Framework (OSF) repository at https://osf.io/cvymj and the Supplementary Material 2.

**Fig. 2:**
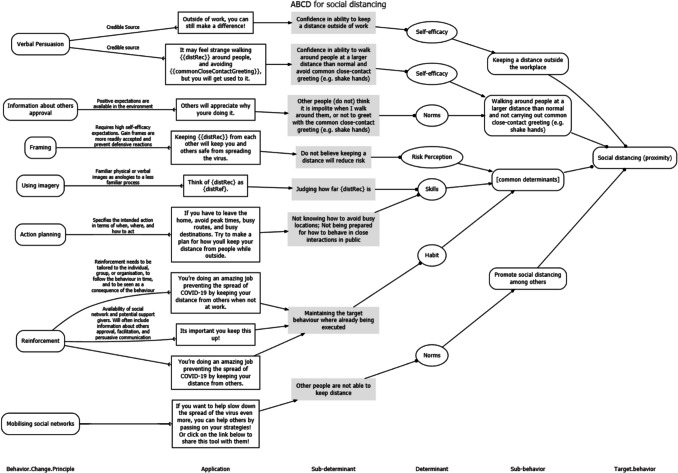
Example of an acyclic behaviour change diagram linking the behaviour social distancing to sub-determinants and example tailored messages in the ‘Application’ frames

Examples of tailored messages can be found in the ABCD diagrams in the column ‘Application’. See Fig. 2 for an example for social distancing, and the OSF sites for self-isolation and hand-washing.

### The determinant mapping questions

As the lack of determinant studies meant that we could not develop evidence-based messages for the intervention part, we decided to ask tool users whether they would be willing to answer items to help improve future versions of the tool. These items were designed to measure behavioural determinants by applying the Reasoned Action Approach (Fishbein & Ajzen, 2010). In total, we developed 276 items (the ‘v1/operationalizations’ directory in the OSF repository at https://osf.io/vkdyt/files/1b1368ae-475f-40cb-824d-69b1d94af585). Tool users could select the number of items they were willing to answer on a scale from 0 to 20 (in steps of 2), and were then presented with a random selection of questions. We used Decentralized Construct Taxonomies specifications to facilitate homogeneous operationalisations in all 22 languages (Peters & Crutzen, 2024; see the ‘v1/operationalizations/dct-files’ directory in the OSF repository for the DCT files) (Supplementary Material 3).

### The your COVID-19 risk tool

The tool, accessible via https://your-covid-19-risk.com, consists of two parts: a static website and a LimeSurvey (LimeSurvey Project Team/Carsten Schmitz, 2020) implementation. The tool is optimised for mobile devices, as many people primarily access the internet using mobile phones (CBS, n.d.; Mitter, 2020; Telemedia, 2020), it does not store any personal data, and it can be used repeatedly. We created a static website (instead of a website using server-side parsed code in, e.g., PHP, Python, or Java) to facilitate free hosting, eliminate the need to implement custom configurations of the web server, and facilitate adaptation of the website. All content was translated from English into 21 languages (making a total of 22 languages) by the international experts participating in the project (forward and back translations were used for most languages). The included languages are Amharic, Arabic, Chinese (simplified and traditional), Dutch, English, French, German, Greek, Hebrew, Hungarian, Indonesian, Italian, Japanese, Korean, Polish, Portuguese (Brazilian and Portuguese), Romanian, Spanish, Turkish, and Urdu.

The static *Your COVID-19 Risk* tool site consists of a homepage, details about the project and project expert volunteers, and pages briefly describing the underlying theory, risk model, and data management. The homepage features a button labelled *‘Find out your risk!*’ that links to LimeSurvey, where tool users answer 10 risk estimation questions (see below) before being sent back to the results page on the static site, which shows them their risk estimate and the tailored behaviour change intervention.

The risk estimation questions assess users’ location (country and, for some countries, also region), age, and gender. The users are then asked to select details about their work environment (i.e., whether they work with patients and vulnerable groups, with children, with co-workers, with the general public, from home, or do not work). Users are also asked about their current daily behaviours (including leaving the house for groceries, to get medical care, exercise, visit family, friends, etc.—multiple options can be selected), and the conditions under which these day-to-day behaviours would change (e.g., if they had COVID-19 symptoms, were in contact with someone who is sick or symptomatic, or if their government imposed further restrictions). Additionally, tool users are asked to identify which of their day-to-day behaviours would change under such conditions (e.g., ‘I would stop visiting family’). Next, users are asked to estimate how often they keep their distance (specifically 1.5 metres) from others in public, identify the situations in which they regularly wash or clean their hands and the procedures they follow to do so. Recommendations for wearing masks were not clearly defined in March 2020, when there were still concerns about supplies for healthcare workers and limited information about or availability of masks.

In the penultimate stage of using the tool, users could opt in to answer additional questions (between 0 and 20 determinant mapping questions) in order to improve the tool. Once they responded to these questions, the survey produced a string encoding the risk estimate and personalised intervention content before redirecting users to their results page on the static site. The results page describes and illustrates the individual user’s ‘*risk estimate*’ and behaviour change intervention (a selection of tailored messages labelled their *‘safety estimate’*).

### Disseminating the your COVID-19 risk tool

The tool was disseminated through our network of expert volunteers involved in the development of the tool. Different dissemination strategies were used in different countries, including through mailing lists and universities and organisations that our team had connections with. In some countries, such as the Netherlands, New Zealand, and Belgium, a comprehensive dissemination strategy was used including social media, press, and radio. The tool advertisements directed interested users to the online survey. The limitation of this dissemination process was that we have not specifically analysed which strategies were most/least effective, and how different dissemination approaches may have impacted sample representativeness. Potentially, there was a self-selection bias, and people who responded to the survey may have been more aware of the COVID-19 risk than the general population.

### Adapting the your COVID-19 risk tool

Adapting the tool requires some familiarity with the technologies used to build it, such as Git, R, and R Markdown, Hypertext Markup Language (HTML), Cascading Stylesheets (CSS), and JavaScript. To adapt the tool, the Git repository hosted at https://gitlab.com/a-bc/your-covid-19-risk can be cloned or forked. The static site is located in the ‘v1/website’ directory, and loads almost all of its content from JavaScript Object Notation (JSON) files in the ‘json’ subdirectory and from ResourceBundle files in the ‘i18n’ subdirectory (‘i18n’ is a numeronym that stands for ‘internationalisation’). The survey questionnaires are located in the ‘operationalizations/limesurvey’ directory: these are tab-separated files in the.txt format that can be imported directly into LimeSurvey. The project relies heavily on a set of R scripts that read data from spreadsheets stored in Google Sheets, create local backups in Microsoft Excel’s.xlsx format, and parse the data to produce the resources that inform the static site and the LimeSurvey part of the tool. These scripts are available in the ‘v1/scripts’ directory.

### Using the your COVID-19 risk data

The Your COVID-19 Risk data are available in a dedicated Git repository that is hosted at https://gitlab.com/a-bc/your-covid-19-risk-data. This repository contains a series of comma-separated values (CSV) files, as well as file manifests in several formats and an R script to import and merge all the data. Each time the data pipeline is run, these files are updated. It is also possible to inspect the analysis files, which are available in the project’s main repository in the ‘v1/scripts’ directory.

## Results

### Using the analysis file for the your COVID-19 risk project

The Your COVID-19 Risk project contains an R Markdown file that analyses the data produced by the tool. The rendered version of this file is hosted by GitLab Pages and accessible via https://your-risk.com/v1-results. In this section, we use brief summaries of the results of these analyses to guide readers through the sections of this file. These results pertain to the full data set (i.e., for all countries combined) and, as such, give a good impression of the tool users, but not of any country, global patterns, or specific time frame.

### Countries

**Fig. 3:**
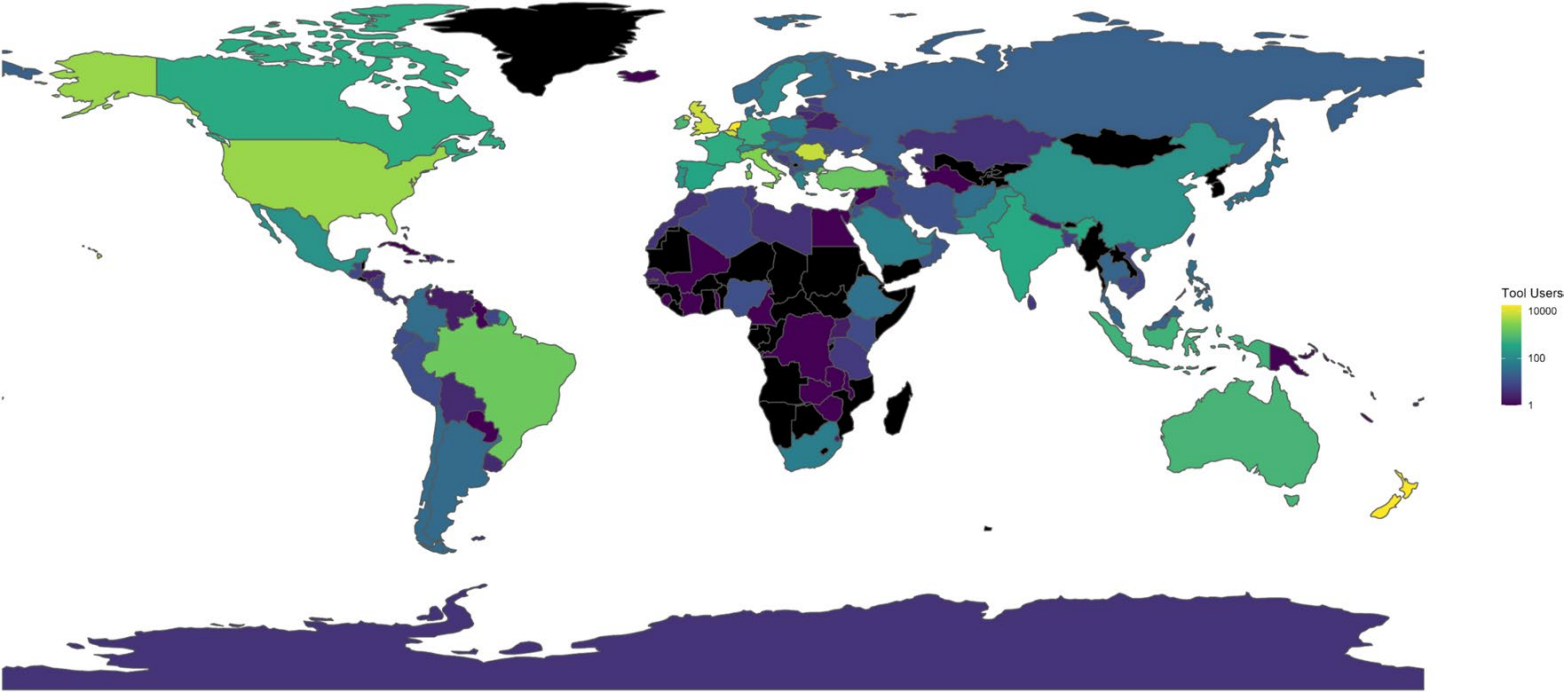
A world map showing the number of tool users across countries

The tool first launched in the Netherlands on May 7th, 2020, and the remaining countries launched using a staggered approach over the following weeks. From this date until June 9th, 2021, 102,909 individuals from 166 countries used the *Your COVID-19 Risk* tool (see the world map of tool users in Fig. 3), of whom 63,850 (from 153 countries) completed the full assessment. Of the remaining 39,059, 27,424 only opened the first page, and 11,635 started but did not complete the risk assessment and did not receive behavioural support. This equates to a 15% drop-out over all countries (i.e., 11,635 out of 102,909 − 27,424). The following countries had 100 or more users who completed the tool: Netherlands (*n* = 15,376), New Zealand (*n* = 14,615), Belgium (*n* = 7,052), United Kingdom (*n* = 6,978), Romania (*n* = 6,187), United States (*n* = 2,998), Italy (*n* = 2,240), Brazil (*n* = 1,415), Turkey (*n* = 1,137), Ireland (Republic) (*n* = 587), Australia (*n* = 523), Indonesia (*n* = 440), Germany (*n* = 385), France (*n* = 340), Canada (*n* = 323), Spain (*n* = 281), India (*n* = 251), Cyprus (*n* = 201), Portugal (*n* = 117), Switzerland (*n* = 112), Mexico (*n* = 109), China (*n* = 104), and Pakistan (*n* = 101). Tool use over time was strongly related to local promotional activities. Usage increased rapidly after the tool launch in May–June 2020, with a few spikes in use during July.

### Characteristics of tool users

Most of the tool users self-reported being in the age category of 40–49 years (20.1%), followed by 30–39 (19.5%), 50–59 (19.4%), 20–29 (17.6%), and 60–69 (14.3%), with 8.9% younger than 20 or 70 or older, and 0.1% declining to disclose their age. Most users identified as female (54.1%) or male (45.2%), with 0.31% identifying as nonbinary or third gender, or providing a self-specified gender.

### Risk estimation questions

In response to the (multiple-choice) question about work-related risks, most tool users indicated they were currently working from home or not working (63.1%), with 28.2% indicating they were (instead or also) professionally in contact with the general public (e.g., in the hospitality or public transport sectors), 6.7% being professionally in contact with patients (e.g., at hospitals, general practitioners, or nursing homes), and 6.1% being professionally in contact with children (e.g., at childcare, primary, or secondary education).

In response to the (multiple-choice) question about the degree to which tool users self-isolated, most tool users indicated that they were currently leaving their house for food, medicine, or healthcare (75.8%), with 70.1% indicating they were (instead or also) walking or exercising outdoors, 52.2% leaving their house for other shops and services, 45.1% mostly staying at home, 35.9% visiting family, and 27.1% visiting friends.

In response to the (multiple-choice) question about what would prompt them to self-isolate more, most tool users indicated that they would self-isolate more if they have symptoms of COVID-19 (85.4%), with 73.3% indicating that they would (instead of or also) self-isolate more if they have been in contact with somebody who is sick or showed symptoms, 65.8% if the government imposed more restrictions, 4.4% if would not consider this at all, and 0.4% specified another reason.

What exactly that means (i.e., self-isolate more) is different for different people. In response to the (multiple-choice) question about what ‘self-isolating more’ would entail for them, 43.4% of tool users indicated that they would stop leaving the house for other shops or services, with 40.6% indicating that they would (instead of or also) stop leaving the house for food, medicine, or healthcare, 34.6% stop outdoor walks or exercise, 31.9% stop visiting family, 25% stop visiting friends, and 3.2% specified another reason.

Regarding keeping their distance from others (the ‘behaviour’ measure used to estimate determinant importance), 0.9% indicated they did this (almost) never; 1.9% rarely; 8.7% sometimes; 23.6% often; 61.5% (almost) always; and 3.5% indicated that they were never in public places.

Most tool users indicated that they washed their hands after they have touched public or frequently touched surfaces (e.g., doorknobs, railings, tables, buttons/switches; 83.4%), with 77.4% indicating that they (instead of or also) washed their hands on entering their home, place of work, or other building they will be spending some time in, 76.6% before they handle or eat food, 65.2% after they have been in direct contact with someone else, 41% after they sneeze or cough, 28.9% before or after they have touched their face, nose, or mouth, and 3.9% indicated they never or only occasionally wash/clean their hands.

Regarding thoroughness, 91.1% of the tool users indicate that they normally use soap or hand sanitiser, with 69.8% indicating that they (instead of or also) wash for at least 20 seconds, 47% clean under their nails and between their fingers, and 1.2% indicate they do not do any of the above.

### Determinant mapping questions

**Fig. 4:**
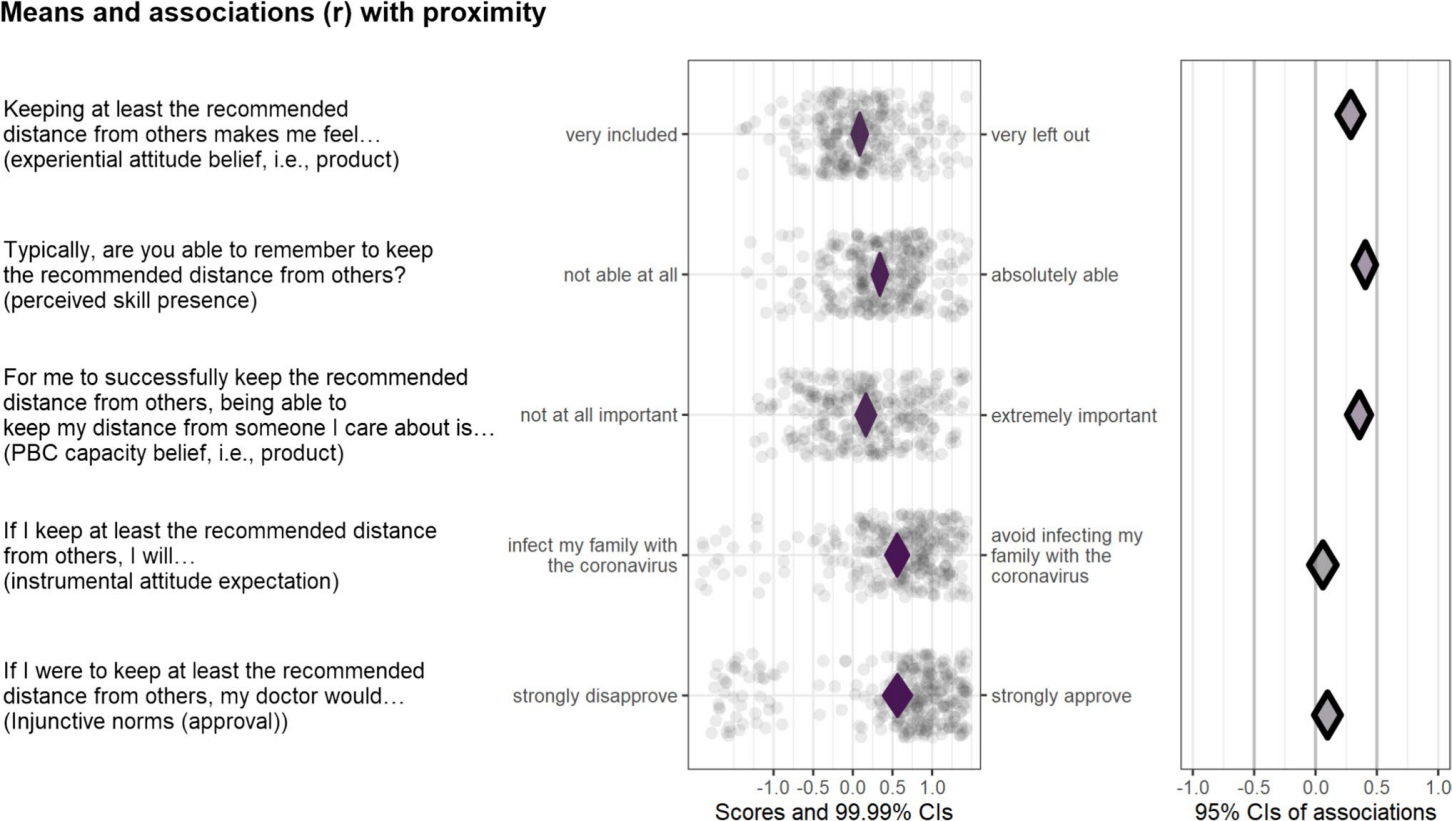
An example of a CIBER (confidence interval-based estimation of relevance) plot, showing three relevant and two irrelevant determinants in the Dutch subsample

Based on users’ responses to the determinant mapping questions (which in this case concerned following their country-specific guidelines for social distancing behaviours), CIBER (confidence interval-based estimation of relevance) plots were created (Crutzen et al., 2017; Peters & Crutzen, 2018). The results of these plots allow the selection of the most relevant determinants, reflecting relevant theoretical, methodological, and statistical considerations, and are available (for each country with enough data) in the Attitude, Perceived Norm, and Perceived Behavioural Control tabs (see https://your-risk.com/v1-results). An example of a CIBER plot is shown in Fig. 4, which illustrates a selection of five sub-determinants: three that were identified as important and two that were not important. In brief, the left panel shows each (sub)determinant’s raw data (i.e., the distribution of sample scores) as well as the 99.99% confidence interval for the mean. The right panel shows the 95% confidence interval of the association with one or more targets—in this specific case, the bivariate correlation with tool users’ self-reported social distancing as measured by one of the risk estimation questions. Whether a sub-determinant is selected as an intervention target normally depends on whether it is bivariately associated with behaviour (or a proxy of behaviour) and on its univariate distribution (indicating room for improvement). High correlations can be driven by only a few data points, so it is possible that sub-determinants with little room for improvement are still associated with the behaviour (Crutzen et al., 2017).

In addition to the CIBER plots, overviews of all sub-determinants are provided in tables in the ‘Overview’ tab. Three overviews are produced: the last one is sorted by the bivariate correlation of each sub-determinant with the target behaviour, and the first two are sorted by two versions of the Potential for Change index (PΔ), which is a quantitative summary of the information in the CIBER plots. The original PΔ was conceptualised to optimise intervention tailoring and improve the prediction of individual-level intervention effectiveness (Knittle & Peters, 2019). For this project, two sample-level variants were computed to facilitate sub-determinant selection.

The first, labelled the *Potential for Change Index 1* (PΔ1), was computed as follows: for sub-determinants with a positive zero-order correlation with behaviour, the sample mean was subtracted from the observed maximum score, and the result was multiplied by the zero-order correlation. For sub-determinants with a negative zero-order correlation with behaviour, the sample mean was subtracted from the observed minimum score, and the result was multiplied by the zero-order correlation.

The second, labelled the *Potential for Change Index 2* (PΔ2), was computed as follows: for sub-determinants with a positive zero-order correlation with behaviour, the sample mean was subtracted from the.95 quantile of the scores, and the result was multiplied by the squared zero-order correlation (i.e., the proportion of explained variance). For sub-determinants with a negative zero-order correlation with behaviour, the sample mean was subtracted from the.05 quantile of the scores, and the result was multiplied by the squared zero-order correlation (i.e., the proportion of explained variance). The second variant effectively takes the 5% trimmed maximum and minimum, rendering it less sensitive to outliers, penalising weak associations with behaviour more severely and decreasing sensitivity to differences between correlations. These differences usually render the second variant more robust across samples, because both trimming data series and squaring correlation estimates decrease the effects of sample idiosyncrasies.

## Discussion

The main aims of developing this tool were to support users to estimate their infection risk, to apply behaviour change theory to provide them with strategies to minimise risk, and to gather data to improve the tool and inform future health promotion interventions and policy during the pandemic. The tool users received their *Risk Estim*ate and their *Safety Estimate*. Both were optimised for the tool users'situation based on their unique responses. The Safety Estimate was developed using acyclic behaviour change diagrams (ABCDs; Peters, 2018) and the determinant mapping questions were used to create CIBER plots to subsequently inform policy and practice initiatives regarding prevention efforts.

We showed that the tool was widely used in several countries around the world, and users utilised this tool to estimate their risk and to receive tailored and personalised information. Tools like the proposed one can be used during future pandemics along with other behavioural interventions (Van Bavel et al., 2020). The novel elements of the tool and the process underpinning tool development, operationalisation and wide-scale deployment formed a pipeline of activities. These collaborative efforts and synchronised processes for designing and deploying a tool like that, creating an open-source data repository, can be replicated by other researcher and practitioner teams. Governments and health agencies can adapt and implement this tool to contain the spread of the virus in the event of a future pandemic (Bonell et al., 2020; Van Bavel et al., 2020).

A similar tool that aimed to assess COVID-19 risk and provide feedback to users has been developed by the World Health Organization (WHO, 2022). This tool asked specific questions relevant to protective and risk-related behaviours, and after each question, gave feedback in relation to that behaviour. For example, tool users were asked about shaking hands and were complimented if they followed best practice. If users indicated that they were not following best practice, they were presented with suggestions on how to improve their behaviour. As with the tool presented here, the aims of the WHO tool included informing decision making, supporting users in protecting themselves and others from infection, and promoting best behavioural practice. The WHO tool was also implemented using open-source LimeSurvey software. A key difference was the number of available languages (22 in our tool versus six in the WHO tool). In addition, the information provided by the WHO tool included main guidelines that were informative for people who were not aware of best practices (keeping distance, hand washing, self-isolating when necessary) but may not be useful for users who were already following best practices; as they may wish to receive more sophisticated and nuanced risk estimates and recommendations. We could not identify the theoretical and evidence bases of the WHO tool: it is not clear which (sub-)determinants they targeted with which messages. Our assessment suggests that the tool only targeted knowledge, as opposed to norms, self-efficacy, or other determinants. In comparison, the tool we described and all underlying materials are open-access and open-source; thus it lends itself well to adaptation by other researchers and organisations. Our tool is transparent in its design and precise in terms of estimating risk and providing tailored behaviour change recommendations. Additionally, the data that we gathered are made publicly available so they can be used by the research community and subsequently by practitioners and policy makers who are responding to current and future pandemics. Tools such as these are needed to support the community in their guideline adherence.

## Strengths and limitations

The tool presented in this article is unique and has several strengths. First, all materials, source code, and design documents are publicly available and align with open science principles to enhance transparency and facilitate adaptation to other contexts or behaviours (Norris & O’Connor, 2019). Second, because the underlying behaviour change theory and assumptions are described in detailed ABCDs and freely available, it is possible for other behavioural scientists to improve the intervention based on new insights into the determinant structures. Third, this tool is based on behaviour change theory and evidence and has the potential to further inform science through the open data that are gathered. Furthermore, behaviour change researchers and practitioners can interpret the results and use these to inform practice and policy. Finally, by being published in multiple languages, this tool is available to communities all over the world, and through its collaborative development considers contexts from many cultures and regions (Reynolds-Cuéllar & Delgado Ramos, 2020). Similarly, the multi-disciplinary nature of the group of expert volunteers and consumer and user involvement (Antonini, 2021) ensured application of a wide array of best practices across different disciplines, such as behavioural science, virology, epidemiology, citizen science, and public health, which allowed us to respect each discipline’s limitations (e.g., the behaviour change intervention was based on psychology, but the risk model was based on virology and epidemiology).

There were two key limitations to the present project. The project was initiated by a large group of expert volunteers from all over the world who responded to a public health emergency of international concern in urgent need of internationally coordinated efforts. As the project progressed, expert volunteers’ time became more limited. This was also the case for the small number of expert volunteers that the project depended on for coordination and production of the mutually aligned instructions. The second limitation was that the interlinking set of software applications used for this project each required specific expertise to connect and manage. This expertise was limited in the team, creating a bottleneck in the project’s progress. The solution to both limitations is gaining resources in the form of financial and practical support. However, funding calls often target either research or software development. Redesigning this project to become research is possible but would introduce considerable delays (requiring, e.g., cross-culturally validated measures and ethical approvals). Similarly, this project did not develop innovative software solutions, but instead combined and used existing applications. Instead of being research or software development, this project’s strength lies in the rigorous application of cutting-edge behaviour change science, combined with a theory- and evidence-based risk model, in a well-designed online application. In the future, similar tools can also employ advances in artificial intelligence and large language models to support translation and data analysis and mining (Kjell et al., 2024; Reddy, 2023; Sonnenburg et al., 2024).

## Conclusions

In this publication, we have described the process and systematic method used to curate a tool to assess COVID-19 risk and provide risk and safety estimates accompanied by persuasive messages. The tool was designed by more than 150 experts including behavioural scientists, psychologists, virologists, methods experts, government representatives, consumers, and other groups. All contributing specialists worked in a coordinated effort to develop a theory- and evidence-based tool in order to collect open-source data to inform future interventions and policies. The database is also open to other researchers who are interested in answering specific research questions related to COVID-19. To advance behavioural science and respond effectively to future pandemics, international coordinated effort is needed. Here, we have presented a tool and infrastructure that has the potential to contribute to slowing down virus spread in case of future pandemics

## Supplementary Information

Below is the link to the electronic supplementary material.

Supplementary file1 (DOCX 13 KB)

Supplementary file2 (DOCX 110 KB)

Supplementary file3 (DOCX 361 KB)

Supplementary file4 (DOCX 21 KB)

## Data Availability

Consistent with the Open Science principles to make all materials, source code, analysis scripts, and data freely available, everything produced in this project is licensed under Creative Commons Attribution licenses or similarly, and available from the project’s GitLab repositories https://gitlab.com/a-bc/your-covid-19-risk and https://gitlab.com/a-bc/your-covid-19-risk-data. The files in both repositories are also available through the project’s main Open Science Framework repository available at https://osf.io/vkdyt/. The frozen registrations in component projects will retain the state of these files regardless of whether the Git repository is updated in the future.
