# Supplementary material for: Collecting behavioural data across countries during pandemics: Development of the COVID-19 Risk Assessment Tool
Source: Behav Res Methods. 2025 Jul 14;57(8):223. doi: 10.3758/s13428-025-02743-x (PMC12259484; doi:10.3758/s13428-025-02743-x)
Supplement: Supplementary file 1 — Supplementary file1 (DOCX 13 KB) [file 13428_2025_2743_MOESM1_ESM.docx]

The supplemental material has been made widely available at the OSF project site at <https://osf.io/vkdyt/>
