# Supplementary material for: Collecting behavioural data across countries during pandemics: Development of the COVID-19 Risk Assessment Tool
Source: Behav Res Methods. 2025 Jul 14;57(8):223. doi: 10.3758/s13428-025-02743-x (PMC12259484; doi:10.3758/s13428-025-02743-x)
Supplement: Supplementary file 2 — Supplementary file2 (DOCX 110 KB) [file 13428_2025_2743_MOESM2_ESM.docx]

# Supplementary Material 1

1. Cite as:
2. Peters, G. Y., Kwasnicka, D., Crutzen, R., ten Hoor, G. A., Varol, T., Berry, E., … & Roozen,
3. S. (Pre-print). Collecting behavioural data across countries during pandemics: Development
4. of the COVID-19 Risk Assessment Tool. <https://doi.org/10.31219/osf.io/b8n5g>

# Your COVID-19 Risk: technical background

1. This appendix contains details about the Your COVID-19 Risk tool. These details
2. pertain both to the process (to make that transparent) and to the technical implementation
3. (to support practitioners and researchers who wish to adapt this Open Source tool).

# Expert Consultation 1

1. Scientific articles on COVID-19 (n=110) were reviewed to determine key risks that
2. were associated with SARS-CoV-2 infection. The list of risks was firstly categorised into
3. behaviour type for (1a) risk behaviours for contracting the virus (i.e., hygiene behaviours,
4. social interaction, transport and travel behaviours, health related behaviours), and (1b) risk
5. behaviours for spreading the virus to others. Overlap between these categories was
6. expected. Secondly, the list of risks was also categorised into other risk characteristics: (2a)
7. other indicators of risk of contracting and spreading the virus (i.e., indicators related to
8. profession, living situation) and (2b) other indicators of risk for developing severe
9. conditions (i.e., general indicators such as health status).
10. The list of risk behaviours and risk characteristics were then presented to 10 virologists
11. and epidemiologists to assess how important these factors were for spreading the SARS-

22

CoV-2 virus. The results were collated and also ranked by specificity (i.e., how many people

23

would be affected). A list of key behaviours and characteristics was defined. Then, vignettes

24

were also assessed with scenarios spanning a range of nationalities and situations

25

illustrating how the virus could be spread. The putative key behaviours and characteristics

26

were formulated into *risk estimate questions* and were answered by experts from the point of

27

view of the person described in the vignettes to ensure they encompassed a range of

28

situations.

29

# Expert Consultation 2

30

In the final tool, the answers to the questions about the key behaviours and risk

31

factors were equated to a level of risk. The risk estimate section of the tool provided the user

32

with a visualized risk estimation, where users' risk was listed as risk to (1) themselves and

33

(2) to others, each was shown in four categories ranging from lowest to highest risk. A

34

survey was created to determine which level of risk (standard to exponential; 1-4) an

35

individual would be at for each of the assessed behaviours and characteristics. Fifty-seven

36

experts including behaviour change specialists (n=29), virologists/epidemiologists (n=11),

37

and medical professionals

38

(n=17) were asked to: (a) indicate the level of risk performance / non-performance of the

39

behaviours / characteristics would result in, and (b) their level of confidence in their

40

estimate (low [weighted as .33], medium [.66] or high [1]). For part (a) the experts could use

41

the initial risk estimates provided by virologists and epidemiologists, their own specialist

42

knowledge, advice from other sources (e.g., government guidelines) or the academic

43

literature

44

(indicating the source they used).

The initial results were weighted by confidence and a sensitivity analysis was conducted to determine whether confidence-based weighting influenced the risk estimates. This was not the case because the highest rated risks were the same as the preliminary

1. survey conducted with virologists and epidemiologists. The results did not differ by field or
2. expertise (virologists or epidemiologists vs other professions) or by source of knowledge;
3. therefore, all the data were used in the risk estimate tool.

# Designing the Risk Estimate

1. The risk estimates were presented in categories and we consulted health literacy
2. experts about the number of categories. Three to five active categories were determined by
3. behaviour change experts to be easily understood by most people. A larger number of
4. categories would be most desirable to track a change in results; however, it was unlikely the
5. tool would be used for this purpose and too many categories can be hard to parse for those
6. with lower literacy or numeracy levels. We wanted to prevent anxiety for those tool users
7. getting the highest level of risk and also prevent a false sense of security for tool users
8. getting the lowest level of risk. Therefore, the tool was designed in such a way that the
9. highest and lowest categories in the visualisation were not mapped onto risk estimate
10. scores, and so could never be highlighted for a tool user (to not induce excessive fear or
11. complacency, respectively). We compared the results for five and six categories (of which
12. three and four would be attainable, respectively). The vignettes used in the initial testing of
13. the risk model were adapted (to ensure all questions could be answered) and used again to
14. answer the risk estimate questions. Two additional scenarios were added (introducing a
15. fictional character Minnie - with the minimal possible risk and Max - with the maximum
16. possible risk) to give the full range of risk scores. Each of the vignettes was scored by two
17. people and the scores on the research estimate questions corresponded to our qualitative
18. assessment of the people in the vignettes. We established that four attainable categories, for
19. a total of six risk levels were suitable.
20. The risk estimate was determined to be due to three factors: ‘risk of getting the virus’
21. (i.e., through proximity or lack of social isolation), ‘risk from not removing the virus’ (i.e.,
22. through lack of handwashing) and other uncontrollable factors (e.g., demographics). The
23. demographic factors (i.e., country, gender, age, occupation) were not controllable, although
24. could affect individuals’ personal risk so the messages were to be presented as ‘*protect*
25. *yourself’* (i.e., if you were at high risk) or ‘*protect others’* (i.e., if you were at low risk). A visual
26. image combining these three factors was considered appropriate to communicate the risk
27. level (e.g., an avatar of someone surrounded by virus) and a speed gauge for the
28. demographic factors to show the reason for changing behaviour. The design choices were
29. discussed within the larger team of volunteers and it was decided that one image for each
30. type of risk was to be presented with a scale to indicate risk category (Figure 3). Six
31. categories were agreed upon (4 active) and captions and text written for each. The proposed
32. materials were piloted with general public representatives to ensure that they were easy to
33. understand and interpret. The materials were adjusted in line with the received feedback.
34. After tool users received their risk estimate, they were presented with what we called
35. the ‘*Safety Estimate’*, which was a tailored minimal behaviour change intervention aiming to
36. support people in three behaviours: keeping sufficient distance from others, self-isolation,
37. and hand hygiene.


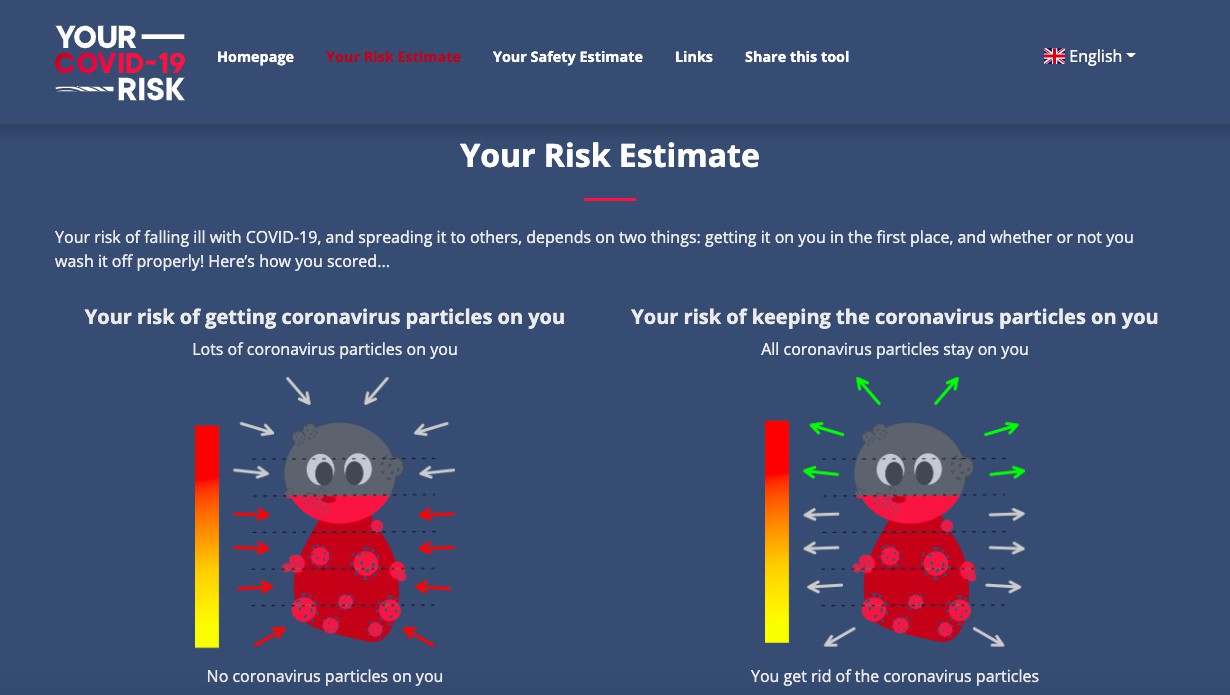
86

87 Figure 3: An example of the graphic representation of risk estimates.
