# Supplementary material for: Collecting behavioural data across countries during pandemics: Development of the COVID-19 Risk Assessment Tool
Source: Behav Res Methods. 2025 Jul 14;57(8):223. doi: 10.3758/s13428-025-02743-x (PMC12259484; doi:10.3758/s13428-025-02743-x)
Supplement: Supplementary file 3 — Supplementary file3 (DOCX 361 KB) [file 13428_2025_2743_MOESM3_ESM.docx]

# Supplementary Material 2

1. Cite as:
2. Peters, G. Y., Kwasnicka, D., Crutzen, R., ten Hoor, G. A., Varol, T., Berry, E., … & Roozen,
3. S. (Pre-print). Collecting behavioural data across countries during pandemics: Development
4. of the COVID-19 Risk Assessment Tool. <https://doi.org/10.31219/osf.io/b8n5g>

# Your COVID-19 Risk: technical background

1. This appendix contains details about the Your COVID-19 Risk tool. These details
2. pertain both to the process (to make that transparent) and to the technical implementation
3. (to support practitioners and researchers who wish to adapt this Open Source tool).

# Designing the intervention

1. The intervention was developed using Acyclic Behaviour Change Diagrams (ABCDs)
2. to optimise alignment between the aspects of human psychology that it targets, the
3. behaviour change principles that were used, and the adequate application of those
4. behaviour change principles. This alignment ensures that the tool offers users what is
5. needed for behaviour change based on a causal-structural chain comprising seven links. The
6. full causal-structural chain of behaviour change is as follows: (1) An effective behaviour
7. change principle is used, and (2) according to the corresponding conditions for effectiveness,
8. (3) the behaviour change principle is applied into an application that targets (4) an
9. important and sufficiently specific sub-determinant, which is a part of (5) a generally
10. defined determinant that determines (together with other determinants), (6) a sub-
11. behaviour, which is a specifically defined behaviour that forms (together with other sub-
12. behaviours), (7) the target behaviour. To illustrate how ABCDs informed the tool
13. development, this concept of the causal-structural chain will first be explained.
14. The chain ends with enactment of the target behaviour. Such target behaviours are
15. typically broadly defined, such as social distancing, self-isolation or hand-washing. Each of
16. these behaviours consists of sub-behaviours. For example, effective hand-washing requires
17. using soap, cleaning hands for at least 20 seconds, and using the right technique. To the
18. degree that each sub-behaviour is determined by individuals’ psychology (as opposed to
19. their environments; together these, by definition, determine behaviour), these influences can
20. be captured in a set of determinants: the psychological constructs that theoretically
21. determine the individual’s behaviour. Determinants can be defined generally (such as
22. ‘attitude’) or at a more specific sub-level (sub-determinants such as ‘perceived probability
23. that using soap will remove the virus from my hands’). Although most psychological
24. research occurs at the general level (that of determinants), intervention providers and health
25. promoters need to communicate specific messages (on the level of sub-determinants).
26. ABCDs therefore distinguish both construct levels.
27. The first three links for the causal-structural chain are the behaviour change principles,
28. the corresponding conditions for effectiveness, and the applications in which the principles
29. are applied. To successfully change people’s psychology —i.e., (sub-)determinants—
30. requires correct application of behaviour change principles: techniques that leverage the
31. evolutionary learning processes through which humans learn (Crutzen & Peters, 2018). Like
32. determinants, those behaviour change principles are defined at a general, abstract level, and
33. have to be translated into specific practical applications for use in interventions. In this
34. translation, the intervention developer needs to keep each behaviour change principle’s
35. conditions for effectiveness in mind: the parameters within which an application can still
36. feasibly engage the underlying evolutionary learning processes from which the behaviour
37. change principle derives its effectiveness (Crutzen & Peters, 2018). One application can
38. contain several behaviour change principles and the same behaviour change principle can
39. be applied in various manifestations in different applications, depending on the available
40. intervention medium, the target population’s culture and other characteristics, and what is
41. known to work best in a given context or for a given behaviour or population. This can
42. quickly become confusing, and ABCDs can facilitate retaining an overview of which
43. behaviour change principles are implemented in which applications and how the conditions
44. for effectiveness are safeguarded.

RUNNING HEAD: YOUR COVID-19 RISK TOOL


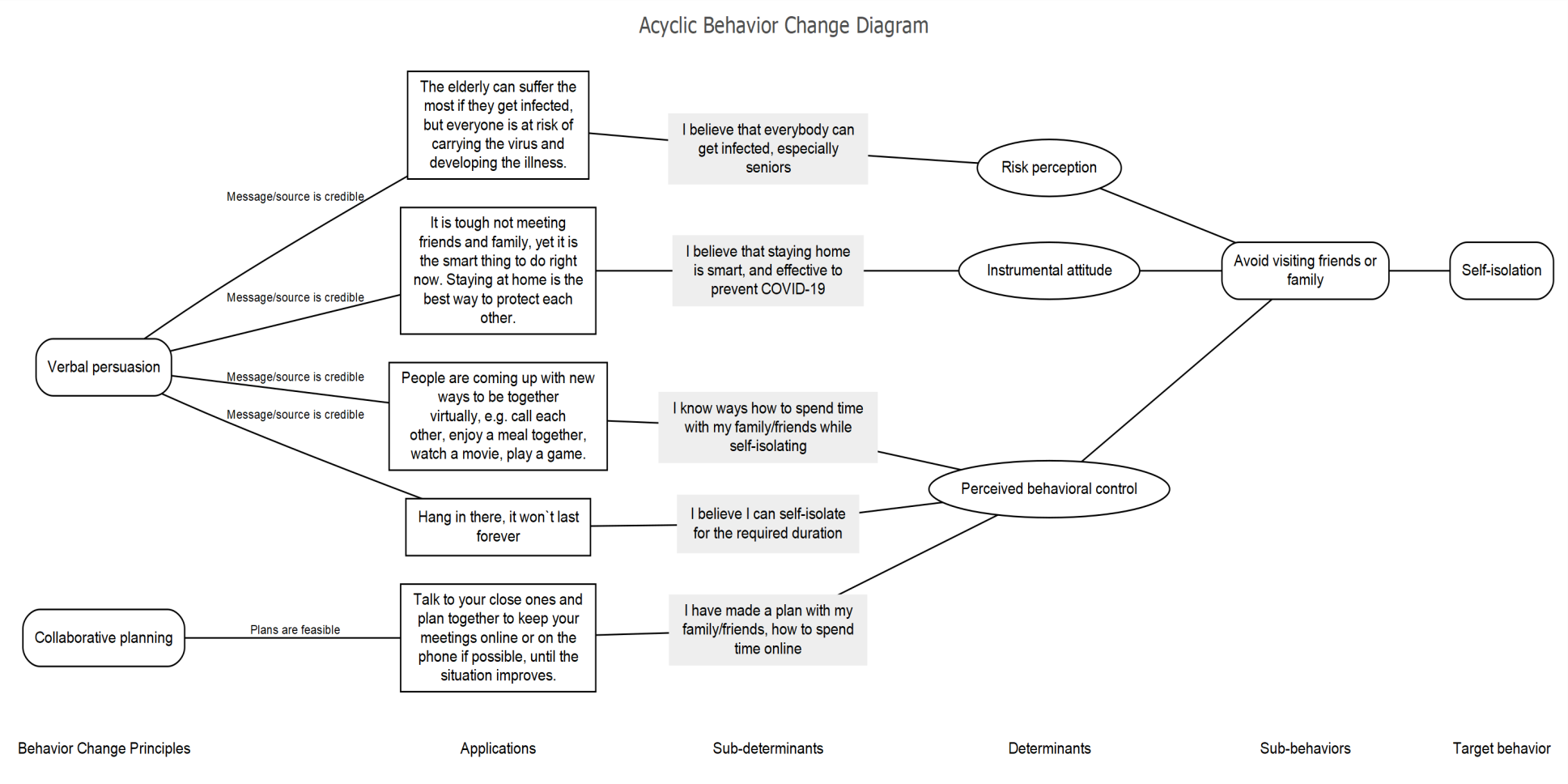
55

56 Figure 4: An illustrative fragment of one of the Acyclic Behaviour Change Diagrams used to develop the intervention 57

RUNNING HEAD: YOUR COVID-19 RISK TOOL

58

1. ABCDs are built from matrices that are commonly stored in spreadsheets where every
2. row represents one causal-structural chain. An ABCD matrix expresses the assumptions
3. underlying the expected effectiveness of any health communication, intervention or
4. campaign. These matrices can be parsed and used to produce the ABCDs (i.e., the
5. diagrams), which are visual representations of the causal-structural chains. A fragment of
6. one of the ABCDs underlying the tool’s intervention is shown in Figure 4, specifically the
7. part of the intervention targeting social isolation in participants who indicated that they still
8. leave the house to visit friends and family. Please note that the context of the intervention
9. (low resources to develop applications; need for applications to be suitable for various
10. countries) limited the possibilities of applications to mostly being text-based messages.
11. ABCDs express exactly which (sub-)determinants an intervention addresses and which
12. behaviour change principles the intervention developers aim to use to change the (sub-
13. )determinants. As such, ABCDs make it possible to spot errors, discuss the choices that were
14. made, and facilitate the process of iteratively improving the tool over time based on new
15. insights about important (sub-)determinants (as well as adaptation of the intervention by
16. others). As we learn more about why people do what they do in terms of COVID-19-related
17. behaviours, we are able to improve the tool to better meet people’s needs. To enable these
18. improvements, we integrated another important aspect into the tool, namely the
19. Determinant Mapping Questions.

78
