# Supplementary material for: Collecting behavioural data across countries during pandemics: Development of the COVID-19 Risk Assessment Tool
Source: Behav Res Methods. 2025 Jul 14;57(8):223. doi: 10.3758/s13428-025-02743-x (PMC12259484; doi:10.3758/s13428-025-02743-x)
Supplement: Supplementary file 4 — Supplementary file4 (DOCX 21 KB) [file 13428_2025_2743_MOESM4_ESM.docx]

# Supplementary Material 3

1. Cite as:
2. Peters, G. Y., Kwasnicka, D., Crutzen, R., ten Hoor, G. A., Varol, T., Berry, E., … & Roozen,
3. S. (Pre-print). Collecting behavioural data across countries during pandemics: Development
4. of the COVID-19 Risk Assessment Tool. <https://doi.org/10.31219/osf.io/b8n5g>

# Your COVID-19 Risk: technical background

1. This appendix contains details about the Your COVID-19 Risk tool. These details
2. pertain both to the process (to make that transparent) and to the technical implementation
3. (to support practitioners and researchers who wish to adapt this Open Source tool).

# The Determinant Mapping Questions

1. When we started on the ABCDs (i.e., started compiling what was known about the
2. most relevant sub-behaviours to distinguish and which sub-determinants and determinants
3. were the most important predictors of each sub-behaviour), it became apparent that almost
4. no information was available on the determinant structures of the target behaviours. Given
5. that the upper bound for an intervention’s effectiveness is formed by how many of the
6. relevant determinants are successfully changed, to improve the tool, we would need to learn
7. about the target behaviours’ determinant structures. We asked tool users whether they
8. would be willing to help improve the tool, and if they were, to answer additional questions
9. about (sub-)determinants, specifically regarding keeping distance from others (we used
10. country-specific guidelines, e.g., 1.5 metres in the Netherlands, using the World Health
11. Organization (WHO) recommendation of 1 metre for countries where country-specific
12. recommendations were unavailable). However, we needed these (sub-)determinants to be
13. standardised across languages as much as possible, without the resources to conduct cross-
14. cultural validation studies. As a solution we used a technology called Decentralized
15. Construct Taxonomies.
16. Decentralized Construct Taxonomies (DCTs) were designed to address hidden
17. heterogeneity in construct definitions and operationalisations in psychological science
18. (Peters, 2020). Such heterogeneity manifests, for example, in the diversity of symptoms
19. measured by depression scales (Fried, 2017b, p. 52), low convergence of theory of mind
20. measures (Warnell & Redcay, 2019), problematic overlap in emotion measures (Weidman et
21. al., 2017), and confusion about the nature and measurement of self-efficacy relative to other
22. determinants (Williams & Rhodes, 2016). Brick et al. recently proposed that a ‘psychological
23. essentialistic bias’ contributes to overreliance on the assumption that psychological
24. constructs are natural kinds (Brick et al., 2020), an assumption that is questioned with some
25. regularity (Fried, 2017a; Gruijters, 2017; Peters & Crutzen, 2017b, 2017a), yet seems hard to
26. eliminate. DCTs provide a tool to make the exact construct definition that is used in a given
27. study explicit and bind that specific definition to instructions for 1) developing
28. measurement instruments for that construct; 2) coding measurement instruments (as used in
29. systematic reviews); 3) eliciting construct content; and 4) coding qualitative data as
30. informative about a construct (both of which are used in qualitative research).
31. First translating a set of DCT specifications for all determinants that we planned to ask
32. tool users about enabled prompting convergence of people’s hitherto implicit ‘personal’
33. construct definitions. However, as DCT specifications derive their value from their
34. comprehensiveness, these could not be developed in the scope of this project. Therefore, we
35. limited the Determinant Mapping Questions to constructs for which we already had a set of
36. DCT specifications available: those defined in the Reasoned Action Approach (RAA).
37. The RAA is a theoretical framework that addresses reasoned action (i.e., planned
38. behaviour) and behaviour change. In line with the RAA, attitudes towards the behaviour,
39. perceived norms, and perceived behavioural control determine individuals’ intentions,
40. while intentions predict human behaviour. The authors of this framework have extensively
41. described how to measure its specific components and constructs (Fishbein & Ajzen, 2010).
42. These descriptions have been further specified in a set of DCTs that were used in this
43. project.
44. Based on these DCTs, a set of template questions was created in English and then
45. translated from English to each of the 22 languages (and back-translated as volunteer
46. capacity allowed). In parallel with this process, lacking the resources to conduct qualitative
47. research to elicit construct content, we ‘self-elicited’ a long-list of potential sub-
48. determinants. This long-list was then curated in five iterative steps to remove duplicates and
49. complement sub-determinants consistent with the RAAs symmetrical construct definition
50. structure (e.g., when an experiential attitudinal expectation was suggested, the
51. corresponding experiential attitudinal expectation was added to the list of questions). The
52. final list contained 276 questions (available through the associated repository); however, due
53. to cultural differences, we allowed translating teams to decide if any question had to be
54. excluded as it was considered sensitive or nonsensical in the local context. The group of
55. volunteers then applied the calibrated question templates from the previous step to these
56. questions to achieve a set of translated questions that, as much as possible, measured the
57. same constructs. These processes (translation of the template questions, construct content
58. elicitation and curation, and translation of the final set of questions) were conducted using
59. Google Sheets, enabling real-time collaboration. These spreadsheets were then imported into
60. R for further processing.

# Project architecture and applications

1. This project was possible because of a large number of Free/Libre and Open Source
2. Software (FLOSS) solutions in combination with free resources graciously offered by
3. LimeSurvey Gmbh, Netlify, and Slack. We only wanted to use FLOSS because we wanted
4. the product of the volunteer effort invested in this project to be freely available for
5. everybody. For example, had we not implemented these Open Science principles and used
6. proprietary software for central components of the project, these products would have been
7. virtually inaccessible to organisations and individuals that were not well funded. In
8. addition, this project had no funding itself, and although many of our volunteers could
9. access proprietary software through their employers, the voluntary nature of their
10. participation meant using employer resources was not a straightforward option.
11. In composing the so-called ‘stack’ or ‘application architecture’ for this project (i.e., the
12. layers of software applications that implement the functionalities that we require), we
13. needed to resolve a core project asymmetry. On the one hand, we needed to collaborate on
14. the development of a system with many interlinking components in many languages. On
15. the other hand, we had a large group of volunteers who were familiar with basic office
16. software such as spreadsheets, but less with version control and collaboration solutions such
17. as Git and programming languages such as R, PHP or JavaScript. As these volunteers would
18. produce and translate a large amount of content in a complex data model, we solved it in
19. the following way.
20. First, we chose two free but proprietary tools to support our internal communication
21. and facilitate collaborating on the same documents. We did not consider this problematic
22. because these tools mostly supported ephemeral interactions, and to the degree that we
23. would need to retain data or products, these could be exported to open formats. Specifically,
24. the first tool we heavily relied on was Slack, an asynchronous communications hub. This
25. enabled us to communicate in sub-groups of arbitrary size and allowed all volunteers to
26. read along when they so desired. The threaded communication format kept the interaction
27. relatively organized (the sheer number of messages was still considered overwhelming by
28. many volunteers, but our distributed roles meant that no single person needed to read
29. everything). The second tool was Google Docs, an online synchronous collaboration office
30. suite, of which we used the word processor application (also called Google Docs) and the
31. spreadsheet application (called Google Sheets). We used the approximately 130 Google Docs
32. documents to provide instructions to the team of volunteers so that their actions were
33. streamlined and different subgroups could work in parallel. We used Google Sheets as a
34. stand-in for a relational database. Whenever structured data needed to be generated, or
35. whenever something needed to be translated, we used Google Sheets for this, with carefully
36. structured spreadsheets that had one worksheet for each language. Because Google Sheets
37. has an Application Programming Interface (API) we were able to directly import these data
38. into R.
39. Second, we used Git to collaborate on the project files with the software developers,
40. designers, and data scientists in the project team. Git is a version control and collaboration
41. solution that has quickly become the standard for software developers and is also becoming
42. more commonplace among scientists: it can be thought of as the tracked changes
43. functionality common in word processors such as LibreOffice Writer or Microsoft Word, but
44. for all files, and with many more options. We used GitLab, a FLOSS development and
45. operations platform augmenting Git’s functionality allowing us, among other things, to
46. effortlessly host a website with the rendered R Markdown files produced by the project.
47. This Git repository also included the main website of the project, which was written mostly
48. by one of our volunteers in HTML, CSS, and JavaScript, using the Bootstrap framework and
49. the jQuery and i18next JavaScript libraries. This static website consisted partly of JavaScript
50. Object Notation (JSON) files with content that was produced by R scripts that imported the
51. raw data from Google Sheets.
52. We used LimeSurvey (LimeSurvey Project Team / Carsten Schmitz, 2020) as an engine
53. to present the tool users with questions and compute their risk estimates. Because the
54. LimeSurvey survey had 543 questions organised in 156 question groups, and all text was
55. translated from English into 21 languages (22 total), it was not feasible to manually enter
56. and update the thousands of text elements (questions, answer options, etc) for each
57. language (e.g., the survey contained 56 298 answer options in total). One of us therefore
58. developed an R package called {limonaid} that we used to programmatically produce a file
59. that LimeSurvey could import. This file was again produced based on processing of data
60. that were imported from Google Sheets. For readers who want to import the file into their
61. LimeSurvey instance: note that because the file is 17.6 megabytes, importing it will likely
62. require updating the server settings in LimeSurvey itself or in PHP or Apache.
63. Another core component was R (R Core Team, 2020), which we used to parse all data
64. and content from the spreadsheets with translations. This effectively means that the data
65. model governing those spreadsheets was hardcoded into the R Markdown source code files.
66. We had R Markdown files to perform the required analyses for the two expert consultations
67. and to compute the Risk Model, to produce 14-day case numbers for each country to assign
68. country-level risk, to produce the ABCDs that formed the intervention, to prepare the JSON
69. files for the static website, to produce the survey file to import into LimeSurvey, and to
70. analyse the tool users’ answers (e.g., generating CIBER plots).
